# Evaluating the effects of technology‐supported music‐making on engagement for people living with dementia in community settings

**DOI:** 10.1002/alz70858_103949

**Published:** 2025-12-24

**Authors:** Jennifer MacRitchie, Justin Christensen, Jonathan M Pigrem, Renee Timmers

**Affiliations:** ^1^ The University of Sheffield, Sheffield, South Yorkshire, United Kingdom

## Abstract

**Background:**

Engaging in music activities may be used as part of dementia care for several meaningful outcomes such as aiding recall of autobiographical memories, providing routes for personal expression, and supporting selfhood and relationships. For three years we have collaborated with community and residential care groups to co‐develop new technologies that make engaging with a range of musical experiences more accessible; our two devices aim to forefront and support the agency of the person living with dementia. The Slider Box (a mixing desk analogy) facilitates playback of pre‐recorded music (Pigrem et al., 2023, 2024). The MMM e‐music box is an electronic version of the mechanical music box where spinning a handle facilitates playback of pre‐recorded music (Christensen et al., 2023). Our research aims to explore whether the engagement of a person living with dementia is affected by the affordances of the distinct musical activity.

**Method:**

We recorded eighty‐three musical engagements from six separate community café visits. Participants engaged in three distinct types of music activity: i) used the slider box to mix stems of familiar music, ii) used the slider box to create new music (audio playgrounds), and iii) used the music box to facilitate playback of familiar music. Participants in the group café setting included people living with dementia (predominantly early to mid stages, with varying types), their carers, volunteers and staff members. Each session was recorded on 360‐degree video (Insta 360 X2), with separate audio (Zoom H1). Each video was rated by two coders using the five engagement dimensions (enjoyment, initiation, involvement, interest and response) of the Music in Dementia Assessment Scale (McDermott et al., 2015) and qualitative assessments of interactions with the device and with others.

**Result:**

Preliminary Bayesian models indicate evidence of an effect of the different technology‐supported music activities on the ratings of MiDAS engagement scores. Qualitative results show a diversity of musical behaviours stemming from participants’ creative choices, such as singing or moving along, talking about memories, or being satisfied or surprised at producing musical output.

**Conclusion:**

Implications point towards using technologies to broaden musical offerings for people living with dementia to satisfy a multiplicity of goals.

